# A unique cause of hemoperitoneum: spontaneous rupture of a splenic hemangiopericytoma

**DOI:** 10.1186/1865-1380-4-13

**Published:** 2011-04-05

**Authors:** Picozzi Stefano Carlo Maria, Massimo Mauri, Luca Carmignani

**Affiliations:** 1 Urology Department, IRCCS Policlinico San Donato, University of Milan, Via Morandi 30, San Donato Milanese, 20097, Milan, Italy; 2 I Surgery Department, IRCCS Policlinico San Donato, University of Milan, Via Morandi 30, San Donato Milanese, 20097, Milan, Italy

## Abstract

Non-traumatic hemoperitoneum may be catastrophic if it is not promptly diagnosed and treated. It is critical to identify this clinical picture and treat any active bleeding. We report the first case in the literature (to our knowledge) of spontaneous hemoperitoneum caused by a cystic splenic hemangiopericytoma. Hemangiopericytomas represent a small subset of soft tissue sarcomas. They rarely originate in the spleen as a primary tumor, with only ten cases having been previously described. The difficulty of predicting the prognosis and clinical behavior of these lesions has been repeatedly stressed. The literature concerning this rare and unusual neoplasm is reviewed.

## Introduction

Non-traumatic hemoperitoneum may occur spontaneously or may be iatrogenic. This uncommon and often unsuspected condition may be catastrophic if it is not promptly diagnosed and treated. The possible causes include hemorrhage from a highly vascular neoplasm, pathological splenic rupture, hemorrhage or rupture of an ovarian cyst, rupture of the gestational sac or other affected anatomic part in an ectopic pregnancy, bleeding from a vascular lesion, anticoagulation therapy, blood dyscrasias, surgery and invasive procedures. It is critical to identify the clinical picture and treat any active bleeding promptly [1]. We report the first case in the literature (to our knowledge) of spontaneous hemoperitoneum caused by a cystic splenic hemangiopericytoma.

## Case Report

A 70-year-old man was admitted to our Urology Department with a 2-month history of left-sided abdominal pain. There was no significant past medical or family history. Previous ultrasonography had shown a 10-cm cystic lesion of the spleen and left hydronephrosis, and a further contrast-enhanced computer tomography evaluation confirmed a 12-cm cystic lesion located at the upper pole of the spleen suspected to be a hemorrhagic cyst and a suspected urothelial lesion of the pelvic ureter causing hydronephrosis.

An electrocardiogram performed at admission documented an unknown atrial fibrillation with high ventricular response. The patient began pharmacological cardioversion with amiodarone, beta-blocking therapy in order to slow the heart rate, and anticoagulation therapy with enoxaparin 12,000 IU/day.

**Figure 1:**
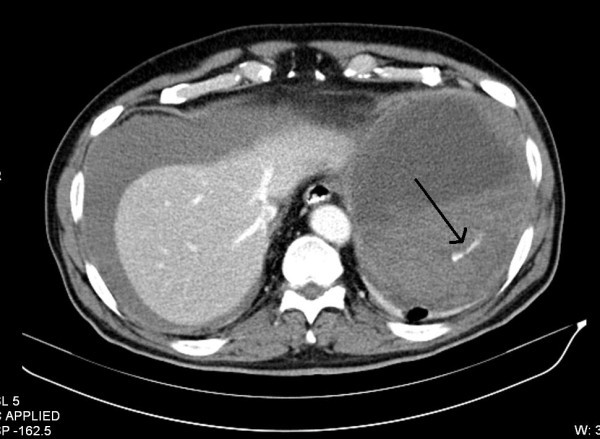
**Abdominal computed tomography scans showing active bleeding in the splenic cystic lesion associated with signs of a recent massive hemorrhage and hemoperitoneum**.

Two days later, the patient developed two episodes of nausea, vomiting, confusion, and sweating associated with abdominal discomfort. The general examination was normal except for pallor, and his hemodynamic parameters were stable with a blood pressure of 110/80 mmHg, pulse of 70/min, and O2 saturation of 99% on room air. Blood tests revealed that his hemoglobin had dropped to 10.2 g/dl from the initial value of 14.5 g/dl. Urgent ultrasonography and computed tomography showed active bleeding in the splenic cystic lesion associated with signs of a recent massive hemorrhage and hemoperitoneum (Figure 1). The patient's condition worsened, and supportive therapy with monitoring was initiated. Central venous access was established in addition to two large-bore catheters in peripheral lines. Fluid resuscitation was initiated with repeated aliquots of 250 ml of Ringer's solution and 6% hydroxyethyl starch 130/0.4 in 0.9% sodium chloride solution, administered with continuous monitoring: systolic blood pressure recovered to 90 mmHg and the radial pulse reappeared.

After stabilization, an emergent exploratory laparotomy was performed, and 3,500 ml of blood was evacuated from the peritoneal cavity. The preoperative hemoglobin was 7.0 g/dl. At surgery, the cystic lesion located at the upper pole of the spleen was found to have ruptured and to be adherent to the diaphragm, colon, and lateral abdominal wall. After splenectomy with excision of the cystic lesion, a drain was inserted and the wound closed. The patient's hemodynamic conditions remained stable, and he was transferred to the intensive care unit where he received a total of 5 units of packed red blood cells and 2 units of fresh-frozen plasma. The postoperative recovery was uneventful. Histological examination of the cystic lesion showed that it was a splenic hemangiopericytoma.

At the 12-month follow-up, computed tomography revealed recurrence of the lesion at the greater omentum, splenic flexure, and stomach. An exploratory laparotomy confirmed the radiological findings, and the greater omentum and lesions were excised.

## Discussion

Hemangiopericytomas constitute a small subset of soft tissue sarcomas. They were first named by Stout and Murray in 1942, and described as distinct vascular soft tissue tumors characterized by groups of endothelial-lined tubes and sprouts, featuring Zimmerman's pericytes [2,3]. Only recently, evolving histopathological concepts and the new 2006 WHO classification of soft tissue sarcomas abandoned this previous vision of a pericyte-derived tumor in favor of a fibroblastic cell origin, integrating this lesion into the family of solitary fibrous tumors [3].

These neoplasms primarily affect adults aged between 20 and 70 years old, with the median age in the 40 s and 50 s. Males and females are affected equally [3-6]. The most common anatomic locations are the lower limbs, axilla, retroperitoneum, pelvic fossa, and head and neck, although hemangiopericytomas have also been described in many other locations such as the lung, breast, peritoneum, liver, pancreas, stomach, greater omentum, mediastinum, bone, inguinal region, uterus, ovary, and vagina [7]. Hemangiopericytomas rarely originate in the spleen as a primary tumor (only ten cases have been described in the international literature), and this presentation was first reported by Guadalajara Jurado et al. in 1989 [8-16]. Splenic hemangiopericytoma is typically asymptomatic and results in splenomegaly. There may be single or multifocal lesions. Given their hypervascular nature, expansive growth, change in the structure of the vessel walls, erosion and necrosis, these lesions can bleed.

Macroscopically, the tumor appears soft and rubbery, and is encapsulated. The average size of such tumors is 6.5 cm [5]. Microscopically, hemangiopericytomas are characterized by well-developed, branching, "stag-horn," thick-walled vessels surrounded by a connective tissue sheath, moderate-to-high cellularity, and a monotonous appearance under light microscopy examination [3]. Imaging studies show a rounded, sharply outlined mass of homogenous density. The typical ultrasonographic picture is a mass with clearly defined margins and a heterogeneous echo pattern that is highly vascularized on color Doppler [17].

On computed tomography, the lesion appears as a well-demarcated, highly vascularized soft tissue mass that can displace adjacent organs [7]. Cystic areas of low attenuation, consistent with necrosis, calcifications, which are frequent in large lesions, and invasion of surrounding structures can be present and are suggestive of a malignant form. Despite improvements in imaging techniques, the differential diagnosis and management of splenic hemangiopericytoma are problematic because of the rarity of the disease. Staging studies include chest, abdominal, and pelvic computed tomography.

The difficulty of predicting the prognosis and clinical behavior of hemangiopericytomas has been repeatedly stressed in the literature. Certain characteristics of the tumor, such as large size (>5 cm), a high number of mitotic figures (>4 mitoses/10 high-powered field), cellular atypia, presence of necrosis, and/or hemorrhage can help to differentiate the malignant form from the benign form [3,5]. The reported rate of metastasis varies significantly from 10 to 60% [3,5,6,18,19]. The pattern of malignant spread is principally hematogenous to the lung, bone, and liver, while lymphatic metastases are uncommon.

Surgical resection, ideally with negative microscopic margins, is the treatment of choice. Local recurrences have been reported in one-third of patients [6]. Oncological treatment standards are hampered by organotropic hemangiopericytomas that mimic benign forms or require emergency operations for bleeding. Radiotherapy reduces local recurrences, as shown for other soft tissue sarcomas, while the role of adjuvant chemotherapy is controversial. The management of local recurrences and metastatic disease is challenging, because no clearly effective therapy exists. In particular, hemangiopericytoma originating in the abdomen behaves aggressively and a careful, life-long follow-up is required for patients because recurrence and metastases can develop after an extended disease-free period.

Written informed consent was obtained from the patient for publication of this case report and accompanying images. A copy of the written consent is available for review by the Editor-in-Chief of this journal.

## Competing interests

Financial or other relationships that might lead to a conflict of interest: none.

## Authors' contributions

The manuscript has been read and approved by all the authors, the requirements for authorship have been met, and each author believes that the manuscript represents honest work.
